# Magnitude and Factors Associated with Research Misconduct at Public University in Ethiopia: A Cross-Sectional Survey

**DOI:** 10.12688/f1000research.159997.1

**Published:** 2025-01-20

**Authors:** Habtamu Belay Hailu, Telahun Teka Wolde, Betselot Yirsaw Wubete, Joseph Ali, Sintayehu Tsegaye Bitew

**Affiliations:** 1 Ethiopian Defense University, College of Health Sciences, Bishoftu, Ethiopia; 2 Addis Ababa University College of Health Sciences, Addis Ababa, Ethiopia; 3 Global Funds Country Coordinating Mechanism of Ethiopia, Addis Ababa, Ethiopia; 4 Johns Hopkins Berman Institute of Bioethics, Baltimore, Maryland, USA

**Keywords:** Research misconduct, Responsible Conduct of Research, Questionable Research Practices, Research Integrity

## Abstract

**Background:**

Research integrity, essential for ethical scientific research, has been inadequately addressed in Ethiopia, resulting in gaps in addressing misconduct like plagiarism, falsification and fabrication.

**Methods:**

An institutional-based cross-sectional study was conducted on a random sample of researchers. Data were collected via a self-administered, structured questionnaire, which was adapted from a similar study. The collected data were analysed using descriptive, bivariate, and multivariable logistic regression.

**Result:**

A total of 244 researchers participated in the study, resulting in an 82% response rate. In our study, 37.7% of participants reported engaging in at least one form of misconduct, 95% CI [31.6%, 44.1%]. Authorship misconduct was the most common form of self-reported misconduct (47.5%), 95% CI [41.1%, 54.0%], followed by fabrication and falsification (40.6%), 95% CI [34.4%, 47.0%]. Publication pressure was significantly associated with research misconduct (AOR = 3.18; 95% CI: [1.02, 9.95]).

**Conclusion:**

Research misconduct has profound implications, compromising the validity of scientific findings and eroding public trust in research. Implementing comprehensive education initiatives on responsible research practices, as well as building an all-encompassing institutional policy, can help to reduce the occurrence of misconduct.

## Introduction

Research misconduct (RM) refers to unethical or dishonest behaviour in the context of academic or scientific research. It encompasses a range of actions that violate the principles of integrity, honesty, and transparency in research. Common forms of research misconduct include plagiarism, fabrication, or falsification of data aimed at misleading or manipulating the scientific community. Making up data or findings and recording or reporting them is defined as fabrication while falsification is changing or eliminating data or results, or interfering with research supplies, tools, or processes in such a way that the study is not properly documented in the research record. Plagiarism is defined as the unauthorized use of another person’s ideas, procedures, outputs, or words. ^1– 4^ RM undermines the credibility of scholarly work, compromises the integrity of scientific findings, and can have far-reaching consequences for the advancement of knowledge and trust within the academic community. ^5^

Deficits in research integrity pose a significant challenge to the credibility and reliability of scientific findings in the field. Despite stringent ethical guidelines and regulations, instances of questionable research practices persist, leading to concerns regarding the accuracy, reproducibility, and transparency of biomedical studies. The ethical conduct of research is guided by key principles, including honesty, objectivity, carefulness, credit, and transparency. Honesty requires researchers to truthfully report data, results, methods, and conflicts of interest in all scientific communications. ^6– 8^ The principle of credit emphasizes giving proper recognition to others’ contributions, preventing plagiarism, and ensuring fair attribution in publications and patents. ^9^ Key factors contributing to research misconduct include pressure from funders, pursuit of recognition, publication pressure, inadequate penalties, lower academic rank, and lack of ethics training. These factors create conditions where ethical violations are more likely to occur. ^2, 10, 11^

Several studies that explore RM, including in some countries in sub-Saharan Africa, have emerged to help increase collective understanding of the phenomenon. According to an article focusing on sub-Saharan Africa, several authors suggest that Low- and Middle-Income Countries (LMICs) may frequently disregard research integrity norms, despite a lack of substantial evidence to support this claim. ^12^ In an exploratory survey of a convenience sample of researchers conducted in Nigeria in 2013, 68.9% reported participation in at least one form of misconduct. ^10^ Similarly, a cross-sectional survey among Kenyan investigators focused on HIV research indicated that 68.3% of respondents engaged in any misconduct. ^13^ These findings from the African context are alarming in their own right and significantly exceed the global prevalence reported in the systematic review and meta-analysis by Xie et al. in 2021, in which the overall prevalence of RM involving at least one misconduct was 2.9% across 42 publications published between 1992 and 2020 across different regions of the world. ^14^

Ethics training is a topic of ongoing debate, with some experts emphasizing its importance in fostering research integrity, ^11, 15, 16^ while others argue that it may not always achieve the desired impact. ^5, 17, 18^ This divergence in views highlights the complexity of the issue. On the one hand, proponents believe that structured training programs can instil ethical principles and improve decision-making in research. On the other hand, critics suggest that the effectiveness of these programs varies, and in some cases, may not be enough to significantly reduce research misconduct. Understanding these differing perspectives is crucial to evaluating the true role of ethics training in promoting responsible conduct in research.

Retractions can serve as a significant indicator of RM, offering insights into unethical research practices. Retraction analyses have shown that research misconduct is a global issue, and Ethiopia is no exception. A study by Rossouw et al. examined retractions between 2014 and 2018 involving African authors or co-authors, finding that biomedical and health sciences accounted for over 60% of the 245 retracted papers. Ethiopia was one of 17 African countries included in the database, with 5 (2.04%) of its authors’ works retracted while neighbouring Kenya accounted for 2.45% of retractions. ^19^ Despite these findings, we did not identify any published empirical studies specifically investigating research misconduct in Ethiopia.

To the best of our knowledge, the magnitude of RM has not been studied among researchers in Ethiopia’s academic and research institutions. To that end, the purpose of the study was to assess the magnitude of RM – as well as associated attitudes and factors – among faculty researchers conducting biomedical and epidemiological studies involving human participants in an academic institution in Ethiopia.

## Methods

### Study design and setting

An institution-based cross-sectional study was conducted to investigate the magnitude, attitudes, and associated factors concerning RM from January to April 2024 among faculty researchers at a public University in Ethiopia.

### Study population

The study included faculty members who were actively involved in health or health sciences research and who had at least one publication.

### Ethical aspects

This study was conducted in accordance with the ethical principles outlined in the Declaration of Helsinki. Ethical approval was obtained from the Addis Ababa University, College of Health Sciences, School of Public Health Institutional Research Ethics Review Committee (IRERC), with approval number SPH/296/2024 and dated 19/01/2024. Written informed consent was obtained from all participants prior to their inclusion in the study.

### Eligibility criteria

• Inclusion criteria: Faculty members with research experience and publications in local and international journals within the last five years were eligible for the study.
• Exclusion criteria: Individuals who had not engaged in research and publication, along with faculty members who were unwilling to participate or did not give consent, were excluded from the data collection.

### Questionnaire

The questionnaire was divided into four sections to collect study participants’ baseline characteristics, general attitudes toward RMC, behavioural influences (factors), and respondents’ self-report of the frequency of their research misconduct practices. Behaviours related to RM and Questionable Research Practices (QRPs) were grouped into five research misconduct composites: circumventing research ethics regulations; fabrication and falsification; plagiarism; authorship misconduct; and conflict of interest.

The questionnaire was adapted from the study on “Reliability and validation of an attitude scale regarding responsible conduct in research” among Middle Eastern researchers. ^20^ Questionnaires were collected in such a way that no third party or even the researchers could link specific respondents to completed questionnaires to ensure complete anonymity.

In this study, “Research Misconduct” encompasses composites such as circumventing research ethics regulations, plagiarism, fabrication, and falsification; while “Questionable Research Practices” are defined as including authorship issues and conflicts of interest composites. Each misconduct composite is defined by specific questions.

### Sample size determination

Based on a margin of error of 5%, a confidence level of 95%, an estimated prevalence rate of 52.8% in the Middle East, ^11^ and a total population size of 836 investigators at the targeted university, we calculated a sample size of 297 participants using Cochran’s formula. ^21^

$$n_{0}=\frac{{\left(Z_{\alpha /2}\right)}^{2}*\left(p\right)\left(q\right)}{{\left(d\right)}^{2}}\;$$ (1)

For this study, sample sizes were calculated for each specific objective, and the largest sample size was used as the final sample size. Using the formula above, the largest sample size, *n*_0_ = 383, was determined using the prevalence of 52.8% for the second specific objective regarding attitude from a study in the Middle East. ^11^

$$n_{0}=\frac{{\left(1.96\right)}^{2}\;\left(0.528\right)\left(0.472\right)}{{\left(0.05\right)}^{2}}=383$$

Since this sample size exceeds 5% of the total population (836 * 0.05 = 42), the final sample size was adjusted using Cochran’s correction formula. ^21^

$$n_{1}=\frac{n_{0}}{1+\frac{n_{0}}{N}}\;$$ (2)

By substituting the values into the correction formula (2) provided above, the minimum sample size (adjusted) was determined to be 263.

$$n_{1}=\frac{383}{1+\frac{383}{836}}=263$$

Bartlett et al. recommended using response rates from previous studies of the same or similar populations to address non-response rates. ^22^ Therefore, adjusting for a response rate of 88.7% from a prior study in Nigeria, ^10^ the final sample size was calculated as n _1_ = 263/0.887 = 297.

### Sampling procedure

**Figure 1.**
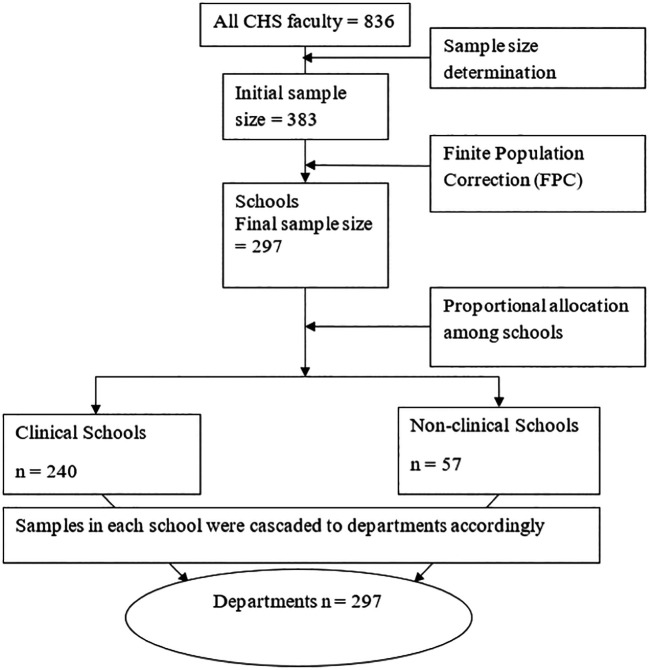
Schematic presentation of sampling procedure.

We used probability sampling approach to select respondents, employing simple random sampling method to ensure random selection and representativeness of our sample. This approach maintained the randomness and representativeness of our sample. The sample size of 297 was distributed across four schools based on the proportionate population size in each of the four disciplines, considering individuals with at least one publication. Each department’s share was calculated by dividing 297 by the total population of 836 and multiplying by 0.35. Study units were then selected from each department using a simple random sampling procedure. This sampling procedure is illustrated schematically in Figure 1.

### Bias

To minimize potential biases in this study on research misconduct, validated tools and metrics were employed to ensure reliable measurement, ^20^ and data collectors were trained to ethically and confidentially collect the data. Confidentiality and anonymity were emphasized to mitigate social desirability bias. Data analysis was conducted objectively using statistical software to ensure accuracy and reduce human error. Efforts were made to avoid confirmation bias by incorporating diverse perspectives in the study framework and seeking evidence that challenged initial hypotheses. Transparent reporting of methodologies, assumptions, and limitations further enhanced the credibility of the research.

### Variables of the study

Independent variables

• **Baseline characteristics**: sex, academic position, highest degree earned, prior training on research ethics.
• **Behavioural influences (Factors)**: pressure from funders, need for recognition, publication pressure, unclear definition of what constitutes misconduct, insufficient censure for misconduct, and financial conflict of interest.

Dependent variables

• **RM**: circumventing RE regulations, plagiarism, and fabrication and falsification
• **QRPs**: authorship misconduct and conflict of interest

### Data analysis

Data from paper questionnaires were collected, entered into Epi-Data 3.1, and subsequently transferred to SPSS version 27. Descriptive analyses summarized demographic frequencies and percentages. Bivariate analysis was utilized to explore relationships between the composite scores of misconducts and factors such as age, gender, prior ethics training, graduate school attended, and academic position.

Respondents were asked about the frequency of various misconduct behaviours, with response options including “Never,” “Once or twice,” or “Three or more times” for self-reported misconduct (RM, QRPs, & Overall RM). To ensure suitable categorization and sufficient data for analysis, these responses were transformed into dichotomous choices: “Never” and “One or more times”. ^23^ Each type of self-reported misconduct was individually measured and categorized into one of the previously mentioned five research misconduct composites. Additionally, the magnitude of self-reported misconduct was calculated for each composite category.

**Figure 2.**
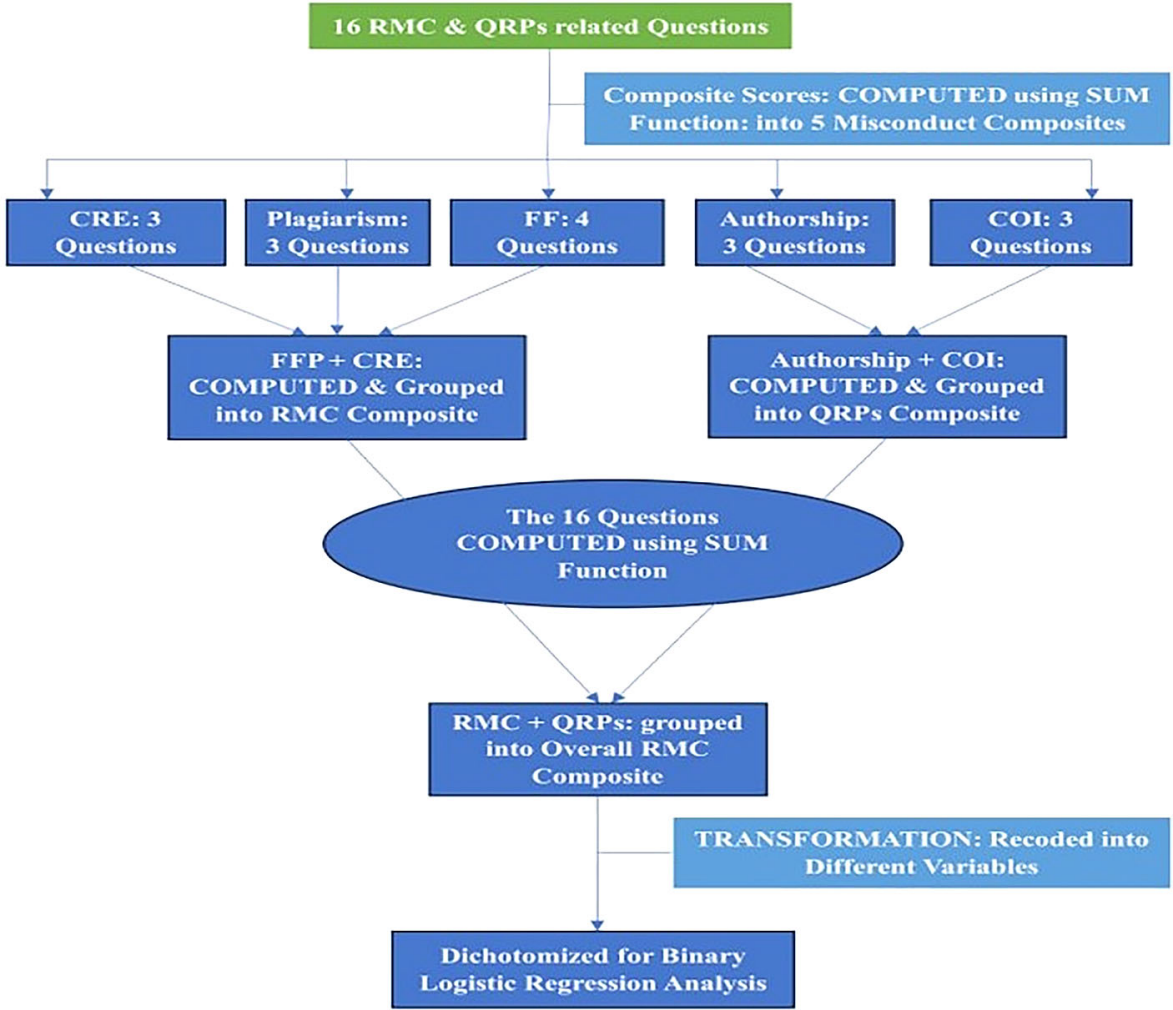
Research misconduct composites transformation procedure.

For composite scores ( Figure 2), multivariable analysis models were constructed. Independent variables identified as significant in bivariate analysis at a p-value threshold of 0.25 were included in the binary logistic regression model. This approach ensures that relevant factors are not overlooked, which can happen when using stricter thresholds like 0.05 in logistic regression modelling. ^24^ A p-value < 0.05 was considered significant for covariates in the final multivariate analysis. Odds ratios (OR) with confidence intervals (CI) and corresponding p-values were calculated.

Regarding ‘attitude’ items, responses were coded on a five-point Likert scale ranging from strongly agree to strongly disagree. Responses of ‘strongly agree’ and ‘agree’ were grouped together as ‘agree,’ while ‘strongly disagree’ and ‘disagree’ were categorized as ‘disagree.’ The mid-point response, ‘neutral,’ was not included in either the ‘agree’ or ‘disagree’ categories. Descriptive analysis then summarized the distribution of responses across these categories. ^11^ Missing data were addressed through mean imputation, replacing missing values with the average of available data for the respective variable.

### Ethical considerations

Prior to filling out the questionnaire, participants were informed of the study’s purpose via a one-page information sheet. Written informed consent was obtained from all participants prior to their participation in the study. Participants were provided with detailed information about the study objectives, procedures, potential risks, and benefits before signing the consent form. This process was reviewed and approved by the Addis Ababa University, College of Health Sciences, School of Public Health Institutional Research Ethics Review Committee (IRERC), with approval number SPH/296/2024 and dated 19/01/2024. The study adhered to the ethical principles outlined in the Declaration of Helsinki.

Due to the sensitive nature of the data acquired and the possibility for dignitary harm, the name of the respondents’ institution was blinded and the names of specific schools were anonymized. The self-administered questionnaire did not include any questions that could reveal participants’ identities, Questionnaires were distributed and collected in person by trained data collectors who explained the study and ensured voluntary participation. After completion, participants placed their questionnaires directly into sealed collection boxes, which were only opened by the researchers after data collection was completed. No identifying information was included on the questionnaires, ensuring that neither the data collectors nor the researchers could link specific responses to individual participants. This process was designed to protect respondent anonymity while encouraging high response rates.

## Results

A total of 297 questionnaires were distributed, with 244 completed, resulting in an 82% response rate. Table 1 shows the demographic and professional characteristics of participants. Almost half (49.6%) ranged in age from 25 to 34 years. Male participants comprised 66.8%. Roughly half (50.8%) were Assistant Professors, and lecturers accounted for 29.1%. Additionally, 49.6% of participants held MD/PhD degrees, while 31.1% held M.Sc./MPH/other master’s degrees. Most participants (90.2%) received their most recent degree from Ethiopian universities. Two-thirds reported prior completion of research ethics training. Most participants (59.0%) had 1-5 years of research experience, while 19.3% had over 10 years. In terms of publication history, 55.7% had published 1-5 papers, and 26.2% had published more than 10. In their most recent publication, 61.1% were co-authors; 38.9% were first authors.

### Respondents’ self-report of behaviours

Table 2 shows the frequency and percentage of occurrences of misconduct composites and associated behaviours reported by respondents. The misconduct composites included circumventing research ethics regulations, fabrication and falsification, plagiarism, authorship misconduct, and conflict of interest. Key findings for the study are summarized as follows.

Concerning the composite circumventing research ethics regulations, several respondents (17.2%) reported not obtaining proper informed consent, 14.8% reported using confidential information about research subjects without authorization, and 12.3% reported conducting research involving human participants without prior approval from a Research Ethics Committee. Regarding plagiarism, 20.9% reported using others’ ideas without credit, 10.7% reported submitting a published manuscript for secondary publication, and 9.4% published others’ results. For fabrication and falsification, 15.2% fabricated data, 14.3% altered data without disclosure, 26.2% selected data to support hypotheses, and 23.0% dropped outliers without mentioning.

Among respondents, reports of authorship misconduct were prevalent, with 37.3% indicating that they gave authorship to those who contributed minimally and 23.0% allowing their name to appear on papers where they contributed little. Non-disclose of conflicts of interest was reported among 17.6% of respondents, 13.1% indicated that they had compromised study rigor as a result of funding pressure, and 7.8% reported altering or suppressing research results inappropriately as a result of funding pressure.

Regarding composites of RM and QRPs, Authorship misconduct emerged as the most prevalent form of research misconduct, with 47.5% of respondents reported involvement (95% CI: 31.6%–44.1%). Fabrication and falsification followed closely, with 40.6% acknowledging participation (95% CI: 34.4%–47.0%). Additionally, 26.6% of respondents reported engaging in plagiarism, while 25.4% reported conflicts of interest, and 29.1% admitted to violating research ethical standards.

### Attitudes regarding selected responsible conduct of research issues

Findings revealed recognition and a strong consensus among participants on the importance of addressing research misconduct, reporting unethical behaviour, declaring conflicts of interest, and mentoring trainees. However, there was some discomfort in discussing ethical issues, which could impede efforts to improve research integrity (see Table 3).

**Table 3.** Attitudes regarding selected responsible conduct of research issues (n = 244).

Question	n (%) Agree	n (%) Disagree
I am concerned about the amount of misconduct	194 (79.5)	50 (20.5)
The responsibility for the scientific integrity of a study lies with the principal investigator only	50 (20.5)	194 (79.5)
Investigators should report instances of research misconduct	228 (93.4)	16 (6.6)
Investigators should declare conflicts of interest to the appropriate officials	230 (94.3)	14 (5.7)
I should monitor my trainees’ work to ensure that they are developing into responsible researchers	235 (96.3)	9 (3.7)
I feel uncomfortable talking with fellow researchers about unethical behavior	54 (22.1)	190 (77.9)

### Perceived behavioural influences on responsible conduct of research

Table 4 shows participants perception toward RCR. Concerning publication pressure, 56.1% considered it moderately influencing while 34.4% considered it strongly influencing. Participants perceived that financial conflicts of interest play a role in RMC, with 46.3% believing it had some influence, while a similar proportion (45.9%) perceived it had strong influence. Regarding pressure from funders, 45.5% of participants perceived it had some influence while 39.3% viewed it as strongly influential. Insufficient censure of misconduct was viewed as an influence in committing research misconduct, with 54.9% believing it had some influence, while 34.4% noting strong influence.

**Table 4.** Behavioural influences in responsible conduct of research (n = 244).

Factors	n (%) No influence	n (%) Influence
Publication pressure	23 (9.4)	221 (90.6)
Financial conflict of interest	19 (7.8)	225 (92.2)
Pressure from funders	37 (15.2)	207 (84.8)
Need for recognition	25 (10.2)	219 (89.8)
Insufficient censure for misconduct	26 (10.7)	218 (89.3)

### Association of attitudes towards certain issues in responsible conduct in research

As Table 5 shows, attitudes towards research integrity are segmented according to ethics training, research experience, and academic status. Results showed that more participants below the rank of lecturer (27.4%) believed that scientific integrity is solely the responsibility of the principal investigator, compared to 16.1% of those above lecturer rank. This indicates a link between academic rank and views on responsibility for scientific integrity. The difference was significant (χ ^2^ = 3.851, df = 1, p < 0.05). Investigators with ethics training tended to endorse the attitude that investigators should report instances of research misconduct more strongly; with a higher percentage (96.3%) agreeing that research misconduct should be reported. The differences were significant (χ ^2^ = 4.906, df = 1, p < 0.027).

In terms of feeling uncomfortable discussing ethical behaviour, academic rank is significantly associated (χ ^2^ = 7.185, df = 1, p < 0.007), as a relatively high percentage (31.6%) of those below lecturer level felt uncomfortable doing so.

### Bivariate analysis of self-reported misconduct ‘one or more times’ within each misconduct composite segmented by potential predictors

A Chi-Square test showed significant associations with a p-value of less than 0.05 (see Table 6). In general, respondents with prior ethics training reported a relatively lower level of self-reported misconduct across all categories when comparing self-reported misconduct with participants without ethics training. Compared to those with ethics training, 34.9% of those without ethics training reported conflicts of interest. The difference was significant (χ ^2^ = 5.290, df = 1, p < 0.021). Across schools, self-reported misconduct varies significantly by affiliation. Respondents from clinical schools, for example, reported greater rates of plagiarism, fabrication and falsification (FF), conflict of interest (CoI), RMC total, and Overall RMC, at 30.9%, 44.5%, 28.8%, 44.0%, and 41.4%, respectively, than those from non-clinical schools. Significant differences were found (χ ^2^ = 7.159, df = 1, p < 0.007), (χ ^2^ = 4.904, df = 1, p < 0.027), (χ ^2^ = 4.528, df = 1, p < 0.033), (χ ^2^ = 8.460, df = 1, p < 0.004), (χ ^2^ = 4.314, df = 1, p < 0.038), respectively.

**Table 6.** Association between self-report of misconduct ‘one or more times’ within each misconduct composite and predictors, 2024 (n = 244).

Misconduct composite	Prior ethics training n (%)		School n (%)		Academic rank n (%)		Research experience n (%)		Highest degree n (%)		Age n (%)	
	Yes (n = 161)	No (n = 83)	Non-clinical (n = 53)	Clinical (n = 191)	Below lecturer ^a^ (n = 95)	Above lecturer ^b^ (n = 149)	1-5 years (n = 144)	>5 years (n = 100)	UG ^c^ (n = 12)	GRAD ^d^ (n = 232)	(25-34) (n = 121)	(35 & above) (n = 123)
CRE ^+^	44 (27.3)	27 (32.5)	13 (24.5)	58 (30.4)	41 (43.2) *	30 (20.1)	48 (33.3)	23 (23.0)	6 (50.0)	65 (28.0)	48 (39.7) *	23 (18.7)
Plagiarism	39 (24.2)	26 (31.3)	6 (11.3)	59 (30.9) *	38 (40.0) *	27 (18.1)	41 (28.5)	24 (24.0)	6 (50.0)	59 (25.4)	35 (28.9)	30 (24.4)
FF ^++^	63 (39.1)	36 (43.4)	14 (26.4)	85 (44.5) *	49 (51.6) *	50 (33.6)	66 (45.8)	33 (33.0)	5 (41.7)	94 (40.5)	56 (46.3)	43 (35.0)
Authorship	77 (47.8)	39 (47.0)	23 (43.4)	93 (48.7)	43 (45.3)	73 (49.0)	64 (44.4)	52 (52.0)	5 (41.7)	111 (47.8)	55 (45.5)	61 (49.6)
CoI ^+++^	33 (20.5)	29 (34.9) *	7 (13.2)	55 (28.8) *	28 (29.5)	34 (22.8)	41 (28.5)	21 (21.0)	8 (66.7) *	54 (23.3)	37 (30.6)	25 (20.3)
RM total	62 (38.5)	33 (39.8)	11 (20.8)	84 (44.0) *	48 (50.5) *	47 (31.5)	64 (44.4) *	31 (31.0)	6 (50.0)	89 (38.4)	56 (46.3) *	39 (31.7)
QRPs total	40 (24.8)	29 (34.9)	10 (18.9)	59 (30.9)	29 (30.5)	40 (26.8)	40 (27.8)	29 (29.0)	6 (50.0)	63 (27.2)	40 (33.1)	29 (23.6)
Overall RM	60 (37.3)	32 (38.6)	13 (24.5)	79 (41.4) *	41 (43.2)	51 (34.2)	54 (37.5)	38 (38.0)	5 (41.7)	87 (37.5)	51 (42.1)	41 (33.3)

* P < 0.05.

^a^ Participant having the rank of lecturer and below.

^b^ Participants having the rank of assistant professor and above.

^c^ Participants holding first degree (Undergraduate).

^d^ Participants holding MSc and above (Graduate).

^+^ Circumventing Research Ethics Regulations.

^++^ Falsification and Fabrication.

^+++^ Conflict of Interest.

The misconduct rates of those above and below the lecturer rank also differed significantly. The study found that 18.1% of participants above lecturer rank self-reported plagiarism, compared to 40.0% of participants below lecturer rank (χ ^2^ = 13.113, df = 1, p < 0.001); Additionally, 51.6% of participants below lecturer rank reported falsification and fabrication, while 33.6% of participants above lecturer reported misbehaviour. Significant differences were seen between these groups (χ ^2^ = 7.085, df = 1, p < 0.008). In terms of RMC-Total, 31.5% of respondents above lecturer rank reported committing RMC, which is considerably lower than the 50.5% of participants below lecturer rank (χ ^2^ = 8.012, df = 1, p < 0.005).

Research misconduct was reported by 44.4% of respondents with less than 5 years of research experience, which was significantly higher compared to the 31.0% reported by those with more than 5 years of experience (χ ^2^ = 3.939, df = 1, p < 0.047). Self-reported research misconduct varies by age, with junior researchers ^25– 34^ more likely to report engaging in multiple types of research misconduct than senior researchers (35+) (χ ^2^ = 4.853, df = 1, p < 0.028). Additionally, significant age differences were observed in bypassing research ethics regulations, with 39.7% of junior researchers admitting to this behaviour, compared to 18.7% of senior researchers (χ ^2^ = 12.005, df = 1, p < 0.001).

### Factors associated with RMC composites

Table 7 highlights several significant predictors of research misconduct and related behaviours. Higher academic rank, older age, and a more responsible attitude toward scientific integrity were associated with a lower likelihood of circumventing research ethics regulations, engaging in plagiarism, and committing fabrication or falsification. Participants who reported feeling high publication pressure or working in clinical schools were more likely to engage in research misconduct.

**Table 7.** Logistic regression results on Research Misconduct Composites (RMC) (n = 244).

Variable	Category	n (%) (Never)	n (%) (≥1 Times)	COR (95% CI)	AOR (95% CI)
Academic Rank vs. CRE	Below Lecturer	54 (31.2)	41 (57.7)		1
	Above Lecturer	119 (68.8)	30 (42.3)	0.33 (0.19, 0.59)	0.47 (0.24, 0.93) *
Academic Rank vs. Plagiarism	Below Lecturer	57 (31.8)	38 (58.5)		1
	Above Lecturer	122 (68.2)	27 (41.5)	0.33 (0.19, 0.60)	0.35 (0.19, 0.65) *
School Affiliation vs. Overall RMC	Non-Clinical	40 (26.3)	13 (14.1)		1
	Clinical	112 (73.7)	79 (85.9)	2.17 (1.09, 4.32)	2.06 (1.01, 4.20) *
School Affiliation vs. Plagiarism	Non-Clinical	47 (26.3)	6 (9.2)		1
	Clinical	132 (73.7)	59 (90.8)	3.50 (1.42, 8.64)	3.46 (1.37, 8.71) *
Publication Pressure vs. Overall RMC	No Influence	19 (12.5)	4 (4.3)		1
	Influence	133 (87.5)	88 (95.7)	3.14 (1.03, 9.55)	3.18 (1.02, 9.95) *
Attitude Towards Declaring CoI vs. Overall RMC	Agree	147 (96.7)	83 (90.2)		1
	Disagree	5 (3.3)	9 (9.8)	3.19 (1.03, 9.83)	4.61 (1.23, 17.29) *
Age vs. CRE	Younger (25-34)	73 (42.2)	48 (67.6)		1
	Older (35 & above)	100 (57.8)	23 (32.4)	0.35 (0.20, 0.63)	0.25 (0.11, 0.56) *
Education Level vs. CoI	Undergraduate	4 (2.2)	8 (12.9)		1
	Graduate	178 (97.8)	54 (87.1)	0.15 (0.04, 0.52)	0.16 (0.04, 0.57) *

1 = indicates reference group,

* Significant at p < 0.05, AOR: Adjusted odds ratio, COR: Crude odds ratio, CRE: Circumventing Research Ethics Regulations, CoI: Conflict of Interest.

Researchers with more than 5 years of experience were more prone to authorship misconduct, while those with postgraduate degrees were less likely to engage in conflicts of interest. School affiliation, particularly clinical vs. non-clinical, also played a role in influencing research misconduct behaviours.

For further details on these associations, including specific odds ratios, confidence intervals, and p-values please refer to the appendix.

## Discussion

The findings of this study underscore the critical importance of maintaining research integrity and the responsible conduct of research within health-related fields. As the first investigation of its kind in Ethiopia, this study revealed significant instances of reported research misconduct, including data fabrication, falsification, and plagiarism, which have the potential to threaten the credibility and reliability of scientific research. By systematically analysing these occurrences, the study highlights the pervasive nature of such practices. These results prompt an urgent call for reinforced ethical standards and robust oversight mechanisms to ensure the integrity of future research endeavours. In what follows, we explore the implications of these findings, propose potential strategies for mitigating research misconduct, and underscore the role of education and policy in fostering a culture of ethical research practices.

As noted by Felaefel et al., comparing misconduct rates across studies is challenging due to differences in study methodologies and operational definitions of misconduct. ^11^ Variations in researchers’ awareness and reporting methods also affect the magnitude estimates of RM and QRPs. Despite these inconsistencies, our study’s findings align with those from studies conducted in Kenya and the Middle East, where similar patterns of research misconduct, such as authorship misconduct and data falsification, have been reported. ^11, 13^

Our study identified a significant magnitude of reported research misconduct among participants, though it was comparatively lower than findings reported in studies from Nigeria, Kenya, and the Middle East. ^10, 11, 13^ The variability in reported rates of misconduct across different studies can be attributed to several factors. Firstly, variations in sample sizes impact statistical power and result variability, potentially influencing the observed magnitude of reported misconduct. Secondly, differences in participants’ levels of research experience and education may affect their understanding of what constitutes misconduct and their susceptibility to engaging in such behaviours. ^4^ This is evident in our study, where more than half of the participants had less than 5 years of research experience and were more likely to have been involved in committing research misconduct compared to their senior colleagues. Finally, the Singapore Statement on Research Integrity highlights the importance of considering cultural and institutional contexts in promoting research integrity and understanding the variability in reporting and actual rates of research misconduct across different settings. ^25^

The multivariable analysis showed that publication pressure was significantly associated with overall research misconduct. Pressure to meet publication targets may lead researchers to report engaging in compromised ethics or unethical practices. This finding aligns with the “publish or perish” culture in academia, which continuously pressures scholars to produce scholarly output. Current academic rules and regulations, such as those outlined by the Ethiopian Ministry of Education, impose publication quotas on academic staff. ^26^ Similarly, the CIOMS guideline on good governance practices for research institutions (2023) affirms that a “publish or perish” mentality increases the likelihood of scientific misconduct. ^27^ Our findings are also consistent with earlier studies that have identified publication pressure as a strong predictor of research misconduct. ^10, 11, 13, 28– 32^

There was a negative association between participants’ age and self-reported involvement in misconduct, suggesting that older participants were less likely to report circumventing research ethics regulations compared to younger ones. This may indicate differences in perceptions of misconduct or reluctance to admit certain behaviours. This finding aligns with previous studies conducted in the Middle East and the U.S.A. ^11, 29^ Conversely, participants with more research experience or publications were twice as likely to report engaging in authorship misconduct as those with fewer years of experience. This suggests that as researchers gain more experience, they may be more likely to report engaging in misconduct, such as authorship wrongdoing. Therefore, it is reasonable to consider that the likelihood of reporting authorship misconduct may be higher among more experienced researchers. ^29^

The present study’s lack of statistical significance between prior ethics training and research misconduct reflects a common pattern in the literature, which has produced conflicting findings about the effectiveness of ethics instruction. According to studies conducted by prominent researchers in the field, research ethics training, including Responsible Conduct of Research (RCR) programs, may not always have the expected impact on research behaviour or ethical decision-making. ^5, 17, 18, 33– 36^ Conversely, other studies have shown that prior ethics training strongly predicts lower research misconduct and benefits trainees. ^11, 15, 16, 37– 40^ The inconsistent findings across studies highlight the complexities of research ethics training and suggest that traditional techniques may not always be adequate in addressing the multidimensional nature of research misconduct. Further exploration of the effectiveness of research ethics training is crucial for understanding the underlying factors contributing to these inconsistencies, particularly given the lack of uniformity in definitions and the significant variability in the quality and nature of such training.

The study may be limited by self-reporting biases, which are common in surveys addressing sensitive topics like research misconduct. Its cross-sectional design restricts the ability to establish causation between factors, necessitating caution in interpreting the findings. Additionally, relying on self-reported data may introduce social desirability bias, leading participants to underreport or misrepresent their involvement in misconduct. As the first study of its kind in the country, there are no comparable studies for reference. Despite these limitations, the study’s findings are crucial for guiding efforts to enhance research integrity and effectively combat misconduct.

Despite the valuable findings of this study, several questions remain unanswered, highlighting the need for further research. Although the study identified factors associated with research misconduct, the interactions and relative importance of these factors are still unclear. Future longitudinal studies could explore temporal associations to better understand the causal mechanisms behind research misconduct. Additionally, while this study used quantitative methods, qualitative methods could provide deeper insights into researchers’ motivations and perceptions of unethical behaviour. The participants were primarily sampled from an academic institution, raising concerns about the generalizability of the findings to other types of research institutions and to other academic institutions. To address this limitation, future studies should include researchers from a wider range of sectors and geographical regions.

## Conclusion

Research misconduct has profound implications, compromising the validity of scientific findings and eroding public trust in research. It distorts the evidence base needed for informed decision-making, potentially leading to harmful policy and clinical practices. Additionally, misconduct can result in significant financial losses and resource wastage, as well as damage the reputations of institutions and individuals involved. Ultimately, it hampers scientific progress and undermines the ethical foundations of research, which are essential for advancing public health and societal well-being. Our findings highlight the critical need for focused interventions to improve research integrity among the health science community at the institution and across the country. Implementing comprehensive education initiatives on responsible research practices, as well as building an all-encompassing institutional policy, can help to reduce the occurrence of misconduct. Our study’s relevance stems from its capacity to advise research and academic institutions about the crucial areas that must be addressed in order to establish an ethical research culture.

Ethics and consent: This study adhered to ethical principles outlined in the Declaration of Helsinki, with ethical approval obtained under approval number SPH/296/2024. Written informed consent was obtained from all participants after providing detailed information about the study’s purpose, procedures, risks, and benefits. To ensure confidentiality, all data were deidentified according to the Safe Harbour method prior to analysis and submission.

For a more detailed explanation of the ethical considerations and consent process, refer to the **Methods** and **Ethical Considerations** sections.

## Authors’ contribution

This study was designed, drafted, and analysed by H.B.H.; T.T.W., B.Y.W., J.A., and S.T.B. made cleaning and revisions on the drafts. All authors reviewed the manuscript.

## Disclaimer

The content is solely the responsibility of the authors and does not necessarily represent the official views of the U.S. National Institutes of Health.

## Data Availability

Zenodo: Magnitude and factors associated with research misconduct at public university in Ethiopia: A cross-sectional survey. ^41^^–^^45^ All data have been deidentified prior to uploading in compliance with the Safe Harbour method to ensure the protection of personal and sensitive information. This project contains the following underlying data:
• SPSS Dataset on Research Misconduct and Questionable Research Practices Description: SPSS file containing raw data used for statistical analysis in the study, https://zenodo.org/doi/10.5281/zenodo.14306711. ^41^
• Table 1: Sociodemographic Characteristics of Participants Description: Data table summarizing the demographic characteristics of the study participants, https://zenodo.org/doi/10.5281/zenodo.14540519. ^42^
• Table 2: Respondents’ Self-Report of Behaviors Grouped Within Defined Misconduct Composites Description: Data table categorizing self-reported behaviors related to research misconduct, https://zenodo.org/doi/10.5281/zenodo.14540618. ^43^
• Table 5: Association of Attitudes Towards Certain Issues in Responsible Conduct of Research and Percentages of Respondents Who Answered “Agree” Segmented by Various Factors Description: Data table analyzing associations between attitudes toward responsible research conduct and respondent characteristics, https://zenodo.org/doi/10.5281/zenodo.14540653. ^44^
• Appendix: Detailed Statistical Data on Predictors of Research Misconduct Description: Supplementary dataset providing detailed statistical analyses of predictors related to research misconduct, https://zenodo.org/doi/10.5281/zenodo.14540669. ^45^ Data are available under the terms of the Creative Commons Attribution 4.0 International license (CC-BY 4.0). The extended data for this study is available at Zenodo with the DOI: https://zenodo.org/doi/10.5281/zenodo.14540865. ^46^ This project contains the following:
• Extended data.docx Description: **Participant Information Sheet:** This document provides detailed information about the study to ensure informed participation. **Consent Form:** This form outlines the consent process and ensures that participants agreed to be part of the study voluntarily. **Questionnaire:** This document contains the survey or data collection tool used in the study. Data are available under the terms of Creative Commons Zero (CC0) license.
