# Supplementary material for: Magnitude and Factors Associated with Research Misconduct at a Public University in Ethiopia: A Cross-Sectional Survey
Source: F1000Res. 2025 Feb 20;14:111. Originally published 2025 Jan 20. [Version 2] doi: 10.12688/f1000research.159997.2 (PMC11868747; doi:10.12688/f1000research.159997.2)
Supplement: Peer review history

# Reviewer report: Reviewer response for version 2

**DOI:** 10.5256/f1000research.178141.r367774

**Published:** 2025-02-27

**Authors:** Wei Feng

**Affiliations:** 1 Wuhan University, Wuhan, China

No further comments.

Is the work clearly and accurately presented and does it cite the current literature?

Yes

If applicable, is the statistical analysis and its interpretation appropriate?

Partly

Are all the source data underlying the results available to ensure full reproducibility?

Yes

Is the study design appropriate and is the work technically sound?

Partly

Are the conclusions drawn adequately supported by the results?

Yes

Are sufficient details of methods and analysis provided to allow replication by others?

Partly

Reviewer Expertise:

research integrity

I confirm that I have read this submission and believe that I have an appropriate level of expertise to confirm that it is of an acceptable scientific standard.

# Reviewer report: Reviewer response for version 2

**DOI:** 10.5256/f1000research.178141.r367775

**Published:** 2025-02-22

**Authors:** Patrick Okonta

**Affiliations:** 1 Obstetrics and Gynaecology, Delta State University, Abraka, Delta, P.O.Box 1031, Nigeria

The authors have responded to my earlier review comments and have appropriately revised the manuscript and offered satisfactory explanations to issues raised in my review comment . The manuscript is approved without reservation.

Is the work clearly and accurately presented and does it cite the current literature?

Yes

If applicable, is the statistical analysis and its interpretation appropriate?

Yes

Are all the source data underlying the results available to ensure full reproducibility?

No

Is the study design appropriate and is the work technically sound?

Yes

Are the conclusions drawn adequately supported by the results?

No

Are sufficient details of methods and analysis provided to allow replication by others?

Yes

Reviewer Expertise:

Research Ethics, bioethics, Maternal health, OBGYN

I confirm that I have read this submission and believe that I have an appropriate level of expertise to confirm that it is of an acceptable scientific standard.

# Response

**Published:** 2025-02-22

**Authors:** Habtamu Belay Hailu

**Affiliations:** Ethiopian Defence University, Ethiopia

Thank you for your time and thorough review of our manuscript. We appreciate your valuable feedback and are pleased that our revisions have addressed your comments satisfactorily. Your insights have helped us improve the quality of our work.

 Thank you again for your support and approval.

# Reviewer report: Reviewer response for version 1

**DOI:** 10.5256/f1000research.175799.r361207

**Published:** 2025-02-08

**Authors:** Patrick Okonta

**Affiliations:** 1 Obstetrics and Gynaecology, Delta State University, Abraka, Delta, P.O.Box 1031, Nigeria

The study has as its objective 'to assess the magnitude of RM – as well as associated attitudes and factors – among faculty researchers conducting biomedical and epidemiological studies involving human participants in an academic institution in Ethiopia.'  The topic is important  especially in the context that it is the first of its kind in Ethiopia. Generally the manuscript is well written and the methods well described to make reproducibility of the study possible. However, the following issues /suggestions need to be addressed

 1. Title.

 I would suggest inserting 'a' in front of 'Public University.

 2. Abstract

       a) The aim of the study should be included either as a subheading or the second sentence in the Background subheading.

      b) The conclusion has to be completely revised as I suggests below

 3. Method

        a) The variable 'Research experience' was measured by the number of years in an academic post. I wonder why the 'number of publications/scholarly works' was not used as a more appropriate marker for research experience?

 4. Results

   a) Table 2 is not in the manuscript.

   b) Page 8- The sentence, 'A Chi-Square test showed significant associations with a p-value of less than 0.05'  has to be expanded to make it complete and self explanatory.

 c) I suggest further analysis on Authorship misconduct should be provided to determine which of the 3 types studied was associated with more years of research experience.

 Conclusion.

 The conclusion as stated does not derive from this particular study or related to the objective of the study as stated by the Authors. The conclusion is too generic and very broad without any specific link to either the results from this study or the stated objectives of the study. It should be completely re-written. In addition, since there was no significant difference in research misconduct between those that had prior training in ethics and those who did not, I wonder the basis for the recommendation of educational interventions in the conclusion

Is the work clearly and accurately presented and does it cite the current literature?

Yes

If applicable, is the statistical analysis and its interpretation appropriate?

Yes

Are all the source data underlying the results available to ensure full reproducibility?

No

Is the study design appropriate and is the work technically sound?

Yes

Are the conclusions drawn adequately supported by the results?

No

Are sufficient details of methods and analysis provided to allow replication by others?

Yes

Reviewer Expertise:

Research Ethics, bioethics, Maternal health, OBGYN

I confirm that I have read this submission and believe that I have an appropriate level of expertise to confirm that it is of an acceptable scientific standard, however I have significant reservations, as outlined above.

# Response

**Published:** 2025-02-10

**Authors:** Habtamu Belay Hailu

**Affiliations:** Ethiopian Defence University, Ethiopia

**Dear Prof. Okonta,**

 We are deeply grateful for your time and insightful comments on our publication, "Magnitude and factors associated with research misconduct at a public university in Ethiopia: a cross-sectional survey." Your observations have given us valuable input on how to improve our study's impact, rigor, and clarity. We address each of your suggestions and describe the upcoming changes below.

 1. Title

 Comment: I would suggest inserting 'a' in front of 'Public University.'

 Response: We appreciate your suggestion. The recommended change will be included in the upcoming manuscript version.

 2. Abstract

 a) The aim of the study should be included either as a subheading or the second sentence in the Background subheading.

 Response: We agree that the abstract should clearly convey the purpose of the research. In the upcoming document, we will make sure that the aim is stated explicitly in the abstract's Background section.

 b) The conclusion has to be completely revised as I suggest below.

 Response: Thank you for the recommendation. We will update the conclusion to reflect the study's findings and make sure it appropriately captures the main findings.

 3. Method

 a) The variable 'Research experience' was measured by the number of years in an academic post. I wonder why the 'number of publications/scholarly works' was not used as a more appropriate marker for research experience?

 Response:

 Thank you for this thoughtful comment. For a number of reasons, we chose to gauge research experience by the number of years spent in an academic position, even though the number of publications or scholarly works is undoubtedly a significant predictor of research production.

 As you perfectly know, research experience is not limited to publication output; rather, it includes more extensive tasks like research design, grant writing, mentorship, peer review, and using ethical judgment. In situations when publishing prospects are impacted by institutional, disciplinary, or systemic considerations, scholars with substantial research experience may have made major contributions to the field without necessarily having a high number of publications.

 Second, publication counts may be a potentially inconsistent indicator of experience due to a variety of external factors that do not necessarily reflect research experience, such as disparities in disciplinary norms (e.g., fields with shorter publication timelines versus those with longer research cycles), institutional support, and research funding availability. Predatory publishing, honorary authorship, and publication pressure can also inflate publication counts without necessarily reflecting true research expertise.

 Last but not least, gauging research experience by years spent in an academic position is consistent with earlier studies looking at research integrity and misconduct. Since academic tenure captures exposure to research-related activities outside of publications, it is frequently used as a stand-in for accumulated experience in studies.

 We do, however, recognize the value of considering the number of publications or academic works as a substitute or supplementary indicator of research experience. We will take this into account when determining the study's limitations and recommend it as an area for further investigation.

 4. Results

 a) Table 2 is not in the manuscript.

 Response **:** We appreciate your observation. Due to its size, Table 2 was excluded from the main text per the editor's recommendation. With a clear reference given in the manuscript's Underlying Data section, it is now accessible through the Zenodo repository. To help readers appropriately, we will make sure that this is stated clearly in the pertinent passages of the text.

 b) Page 8 – The sentence, 'A Chi-Square test showed significant associations with a p-value of less than 0.05' has to be expanded to make it complete and self-explanatory.

 Response: We agree that further clarification is necessary. We will revise this section to provide a more detailed explanation of the statistical analysis and results.

 c) I suggest further analysis on authorship misconduct to determine which of the three types studied was associated with more years of research experience.

 Response: In response to your recommendation that authorship misconduct be further examined in relation to research experience, we performed a binary logistic regression analysis to investigate the relationship between research experience and the three distinct types of authorship misconduct evaluated in our study. The findings showed that, when examined as a composite measure, overall authorship misconduct demonstrated a significant relationship with research experience, but none of the individual types of authorship misconduct demonstrated a statistically significant association with research experience.

 This result draws attention to crucial factors pertaining to the statistical model's type and the data's structure. First, since certain types of authorship breaches may be context-dependent or rare, the lack of significance in individual models may be explained by the statistical power limitations of examining each type of misbehavior independently. On the other hand, the composite measure combines several aspects of authorship misbehavior, which makes the dataset more variable and enhances the model's capacity to identify a general correlation with research experience.

 Furthermore, this finding might be indicative of a larger pattern in behavior, where exposure to or participation in various types of authorship misconduct is influenced by research experience rather than just one type. A larger chance of coming across or taking part in several types of authorship misconduct may result from the more complicated authorship negotiations, institutional pressures, and collaborative dynamics that experienced researchers may confront. Research experience may determine overall authorship practices rather than individual transgressions, as indicated by the substantial connection seen in the composite measure.

 Additionally, logistic regression makes the assumption that the log-odds of the result and the predictor variable have a linear connection. Research experience and particular types of authorship misconduct may have a non-linear relationship or be impacted by unquantifiable moderating factors like the field of study, research culture, or institutional policies. The composite score may more accurately reflect the underlying relationship between research experience and authorship integrity overall since it covers a larger spectrum of misbehavior behaviors.

 We value your suggestion since it has helped us develop a deeper comprehension of the connection between authorship dishonesty and research experience.

 5. Conclusion

 Comment: The conclusion as stated does not derive from this particular study or relate to the objective of the study as stated by the authors. The conclusion is too generic and very broad without any specific link to either the results from this study or the stated objectives of the study. It should be completely rewritten. In addition, since there was no significant difference in research misconduct between those that had prior training in ethics and those who did not, I wonder the basis for the recommendation of educational interventions in the conclusion.

 Response: Your critical evaluation of the conclusion section is greatly appreciated. To make sure it directly reflects the aims and findings of the study, we will carefully edit it. In addition, we will reconsider the rationale behind suggesting educational interventions in view of the findings.

 We appreciate your insightful comments and will take the recommendations into consideration. Our manuscript's impact, rigor, and clarity will all be improved by these modifications. We appreciate you taking the time and using your expertise to review our work.

# Reviewer report: Reviewer response for version 1

**DOI:** 10.5256/f1000research.175799.r362165

**Published:** 2025-02-07

**Authors:** Wei Feng

**Affiliations:** 1 Wuhan University, Wuhan, China

Thank you for the opportunity to review this article. This cross-sectional study investigates the extent of research misconduct (RM) among researchers in Ethiopia’s academic and research institutions using a structured questionnaire. Some findings are particularly intriguing, such as the lack of significant statistical correlation between ethics education and RM. However, I have three primary concerns I would like to discuss with the authors.

 Firstly, the authors examined behavioral influences on responsible conduct in research and explored factors associated with RM composites. While the selected factors appear relevant to RM, the authors do not delve deeply into why these factors may influence RM. This oversight may represent a limitation of the quantitative approach and raises questions for scholars engaged in qualitative research. Therefore, I recommend that the authors provide a more comprehensive explanation of these influencing factors, taking into account the educational and cultural context of Ethiopia, as well as relevant existing theories.

 Secondly, as noted in the Discussion Sec., the findings from Ethiopia align with those from Kenya and the Middle East. Readers may be interested in understanding the uniqueness and broader applicability of this study. The research's distinctiveness should not be solely attributed to the choice of Ethiopia as the research site. This concern is related to my first point regarding the investigation of established factors influencing RM, as the significance of the research appears less pronounced. I suggest that the authors further explore the uniqueness of this study and its potential broader applicability in future research.

 Thirdly, the article exhibits certain limitations in research methodology, particularly concerning the formulation of research hypotheses. The manuscript lacks a dedicated literature review section; instead, it briefly presents current research on RM in the Middle East within the Introduction Sec. This approach does not adequately develop research hypotheses by considering existing literature, which should be followed by validation through the questionnaire. I recommend that the authors enhance the methodological rigor of this cross-sectional study by incorporating a more thorough review of relevant literature to inform the formulation of their research hypotheses.

Is the work clearly and accurately presented and does it cite the current literature?

Yes

If applicable, is the statistical analysis and its interpretation appropriate?

Partly

Are all the source data underlying the results available to ensure full reproducibility?

Yes

Is the study design appropriate and is the work technically sound?

Partly

Are the conclusions drawn adequately supported by the results?

Yes

Are sufficient details of methods and analysis provided to allow replication by others?

Partly

Reviewer Expertise:

research integrity

I confirm that I have read this submission and believe that I have an appropriate level of expertise to confirm that it is of an acceptable scientific standard, however I have significant reservations, as outlined above.

## References

[1] : Beyond the traditional: Extending academic libraries’ roles in research integrity based on the causes of research misconduct. *The Journal of Academic Librarianship* .2024;50(3) : 10.1016/j.acalib.2024.102877 10.1016/j.acalib.2024.102877

[2] : Causal factors implicated in research misconduct: evidence from ORI case files. *Sci Eng Ethics* .2007;13(4) : 10.1007/s11948-007-9045-2 395-414 10.1007/s11948-007-9045-2 18038194

[3] : Exploring Scientific Misconduct: Isolated Individuals, Impure Institutions, or an Inevitable Idiom of Modern Science?. *Journal of Bioethical Inquiry* .2008;5(4) : 10.1007/s11673-008-9113-6 271-282 10.1007/s11673-008-9113-6

# Response

**Published:** 2025-02-10

**Authors:** Habtamu Belay Hailu

**Affiliations:** Ethiopian Defence University, Ethiopia

**Dear Ms. Feng,**

 We greatly appreciate your time and critical input on our publication, "Magnitude and factors associated with research misconduct at a public university in Ethiopia: a cross-sectional survey." Your contributions are crucial in improving the clarity, rigor, and contextual relevance of our research. Below, we present a point-by-point response to your comments and detail how we plan to resolve them in the upcoming updates.

**Comment 1: Exploration of Factors Influencing RM**

*"The authors examined behavioral influences on responsible conduct in research and explored factors associated with RM composites. While the selected factors appear relevant to RM, the authors do not delve deeply into why these factors may influence RM. This oversight may represent a limitation of the quantitative approach and raises questions for scholars engaged in qualitative research. Therefore, I recommend that the authors provide a more comprehensive explanation of these influencing factors, taking into account the educational and cultural context of Ethiopia, as well as relevant existing theories."*

**Response:**

 Thank you for your thoughtful feedback. We realize the necessity for a more in-depth examination of the mechanisms underpinning chosen factors' influence on RM. In next versions, we aim to expand the Discussion Section to investigate how research ethics training, institutional rules, and individual ethical views impact RM.

- Use behavioral and ethical theories, such as Rest's Four-Component Model of Moral Behavior and the Theory of Planned Behavior, to establish a theoretical basis for these influences.
- Contextualize our findings in Ethiopia's research and higher education landscape, including resource restrictions, academic pressure, and institutional control.
- Suggest qualitative studies to better understand the motivations and pressures shaping RM, as quantitative approaches may not fully capture these dynamics.

**Comment 2: Uniqueness and Broader Applicability of the Study**

*"As noted in the Discussion Sec., the findings from Ethiopia align with those from Kenya and the Middle East. Readers may be interested in understanding the uniqueness and broader applicability of this study. The research's distinctiveness should not be solely attributed to the choice of Ethiopia as the research site. This concern is related to my first point regarding the investigation of established factors influencing RM, as the significance of the research appears less pronounced. I suggest that the authors further explore the uniqueness of this study and its potential broader applicability in future research."*

**Response:**

 Thank you for your insightful feedback. We intend to:

- Clearly explain the reasoning behind choosing Ethiopia as the study setting, beyond geographic interest, by highlighting important contextual factors like Ethiopia's growing higher education sector, variations in research integrity policies, and differences in institutional oversight. This will help to strengthen the discussion of our study's uniqueness and wider implications.
- Highlight Ethiopia's distinctive contributions by going into detail about how its ethical, regulatory, and educational contexts differ from those of Kenya and the Middle East.
- By relating our findings to worldwide research integrity issues, especially in low- and middle-income countries (LMICs), where comparable systemic problems may affect RM, we can bolster the conversation about the findings' wider applicability.
- Examine the possibility of doing cross-country comparative research in the future to learn more about the common and distinctive elements affecting RM in different settings.

**Comment 3: Literature Review & Hypotheses Development**

*"The article exhibits certain limitations in research methodology, particularly concerning the formulation of research hypotheses. The manuscript lacks a dedicated literature review section; instead, it briefly presents current research on RM in the Middle East within the Introduction Sec. This approach does not adequately develop research hypotheses by considering existing literature, which should be followed by validation through the questionnaire. I recommend that the authors enhance the methodological rigor of this cross-sectional study by incorporating a more thorough review of relevant literature to inform the formulation of their research hypotheses."*

**Response:**

 We appreciate your insightful comments. We take note of your comment that there isn't a specific literature review section. However, a separate section for the literature review is not required nor provided by the journal's formatting guidelines. Rather, we have adhered to the journal's structure, which incorporates pertinent literature into the Introduction section.

 The following actions have been made to address this concern, even though the research has already been completed, and the hypotheses cannot be changed retroactively:

1. **Strengthening the Literature Review:** In order to strengthen the theoretical underpinnings of our work, we have extended our review of pertinent literature in the introduction and discussion parts. This comprises supplementary sources that enhance the importance of our research inquiry and the rationale behind our hypotheses.
2. **Clarifying the Rationale for Hypotheses:** To clearly show how the body of existing literature influenced the development of our hypotheses, we have updated the introduction. This ensures a clear articulation of the theoretical and empirical foundation for our research.
3. **Addressing Methodological Rigor in the Discussion Section:** We acknowledge the shortcomings of our first literature review and have included a discussion of how a more thorough theoretical framework could be incorporated into future research to further improve the study design.

Key concepts pertaining to research misconduct (RM), its different manifestations, contributing factors, global and regional prevalence (including studies from sub-Saharan Africa), and gaps in the literature—specifically, the lack of empirical studies on RM in Ethiopia—are covered in our Introduction. Additionally, we have added the Institutional Theory of Ethics and the Theory of Planned Behavior (TPB) to improve the methodological rigor of our cross-sectional study and overcome the inherent limitations of our quantitative approach. In order to make sure that our study hypotheses are supported by well-established theoretical and empirical foundations, this inclusion was led by pertinent literature.

 This strategy complies with the journal's format while guaranteeing that the background, current literature, and theoretical foundations are adequately covered.

 We value your opinions and are willing to make any necessary revisions to the Introduction to improve its cohesion and clarity. If you have any specific recommendations for how the literature should be included into the current framework, do let us know.

 Thank you for your insightful comments. We look forward to making the necessary revisions to our text and value any additional advice along the way.
